# Twelve-Year Progression of Osteochondral Lesions of the Talus Observed Using Magnetic Resonance Imaging

**DOI:** 10.3390/diagnostics14121251

**Published:** 2024-06-13

**Authors:** Bradley J. Lauck, Isabel Shaffrey, Albert T. Anastasio, Conor N. O’Neill, Andrew E. Hanselman, Samuel B. Adams

**Affiliations:** 1 University of North Carolina School of Medicine, Chapel Hill, NC 27599, USA; 2 Duke University School of Medicine, Durham, NC 27708, USA; 3 Department of Orthopaedic Surgery, Duke University Health System, Durham, NC 27705, USA

**Keywords:** OLT, osteochondral lesion, talus, osteoarthritis, imaging, progression

## Abstract

Osteochondral lesions of the talus are common injuries that are most often the result of trauma. The natural progression of osteochondral lesions is not well understood. It is still unclear which lesions eventually lead to joint degeneration and osteoarthritic changes and if the treatment method affects the progression. The existing literature surrounding this topic is sparse, with inconsistent findings. The presented images are taken from a 72-year-old man with bilateral osteochondral lesions of the talus. To our knowledge, this is the first published series of images illustrating the natural progression of a patient with bilateral osteochondral lesions of the talus over a 12-year time period.

**Figure 1:**
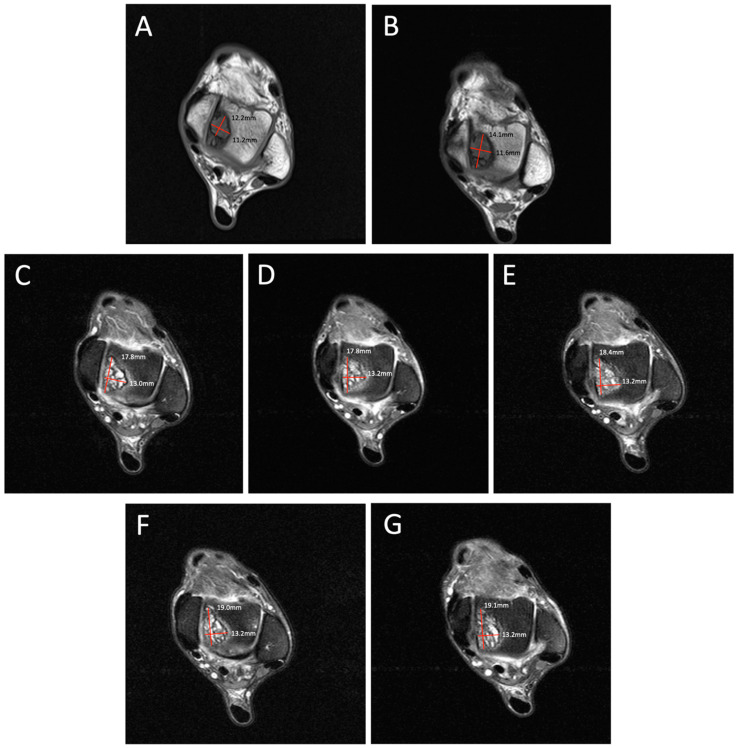
Degenerative progression of the left medial osteochondral lesion outlined in red on axial MRIs from 2009 to 2021. The timeline of lesion progression includes MRI scans from 2009 (**A**), 2011 (**B**), 2016 (**C**), 2017 (**D**), 2018 (**E**), 2019 (**F**), and 2021 (**G**). This axial view demonstrates the osteoarthritic progression of the lesion from 12.2 mm × 11.2 mm in 2009 to 19.1 mm × 13.2 mm in 2021. Patient Case Report: A 72-year-old male presented to the outpatient foot and ankle clinic with a 16-year history of bilateral ankle pain. The patient’s symptoms first began in 2007 following an exercise-induced injury that was left untreated. The patient described his pain as an intermittent waxing and waning sensation of dull pain and aches. Physical examination demonstrated tenderness localized to the anterior aspect of both ankles. Ankle movements were free and without pain. Stability testing revealed mild increased laxity with anterior drawer and talar tilt. He had no other significant past medical history that would contribute to his orthopedic condition. Following his injury, the patient underwent bilateral ankle magnetic resonance imaging (MRI) beginning in 2009, which revealed osteochondral lesions of the talus (OLTs) in both ankles. The patient continued to receive serial ankle MRIs over the following years to monitor the course of the osteochondral lesions over time, totaling seven MRI scans between 2009 and 2021 (Figure 1, Figure 2, Figure 3, Figure 4, Figure 5 and Figure 6). These serial images demonstrated large medial osteochondral lesions in each ankle that showed progressive growth in diameter over time. Though imaging has revealed the gradual growth of the lesions since their onset, the patient has remained relatively asymptomatic. The patient describes his symptoms as dull, intermittent pain that has not had a significant impact on his activities of daily living. However, the patient has had some modifications to his physical activities, including modifying his exercise from running to walking. Overall, the patient remains physically active and reports using an elliptical frequently with minimal pain. Given the patient’s functional abilities and relatively minimal symptoms, the decision was made to continue treatment through non-operative interventions, including monitoring the lesions through imaging and implementing rigid orthotics in his shoes for improved ankle stability. In the presented case, we illustrate the progression of bilateral OLTs over a 12-year time period. The left OLT in our patient progressed from 12.2 × 11.2 mm in 2009 to 19.1 × 13.2 mm in 2021.

**Figure 2:**
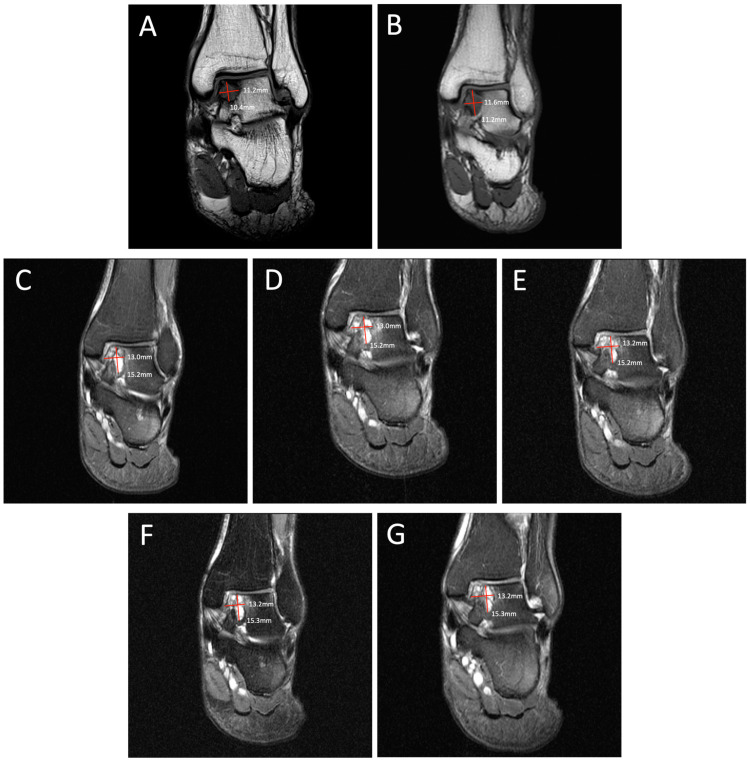
Degenerative progression of the left medial osteochondral lesion outlined in red on coronal MRIs from 2009 to 2021. The timeline of lesion progression includes MRI scans from 2009 (**A**), 2011 (**B**), 2016 (**C**), 2017 (**D**), 2018 (**E**), 2019 (**F**), and 2021 (**G**). Over 12 years, the lesion progressed in size from 11.2 mm × 10.4 mm to 13.2 mm × 15.3 mm on this coronal MRI.

**Figure 3:**
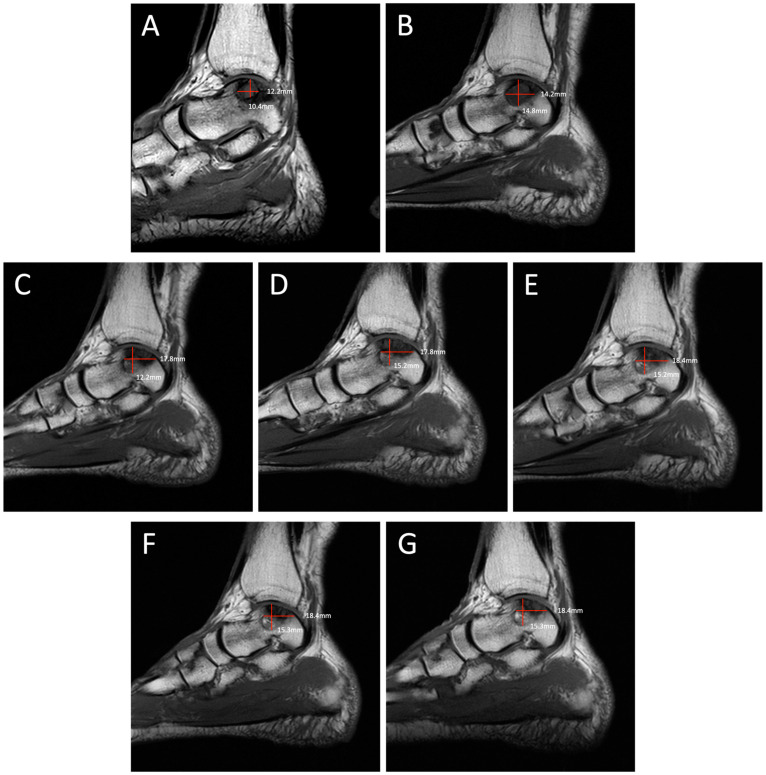
Degenerative progression of the left medial osteochondral lesion outlined in red on sagittal MRIs from 2009 to 2021. The timeline of lesion progression includes MRI scans from 2009 (**A**), 2011 (**B**), 2016 (**C**), 2017 (**D**), 2018 (**E**), 2019 (**F**), and 2021 (**G**). This sagittal view demonstrates the osteoarthritic progression of the lesion from 12.2 mm × 10.4 mm in 2009 to 18.4 mm × 15.3 mm in 2021.

**Figure 4:**
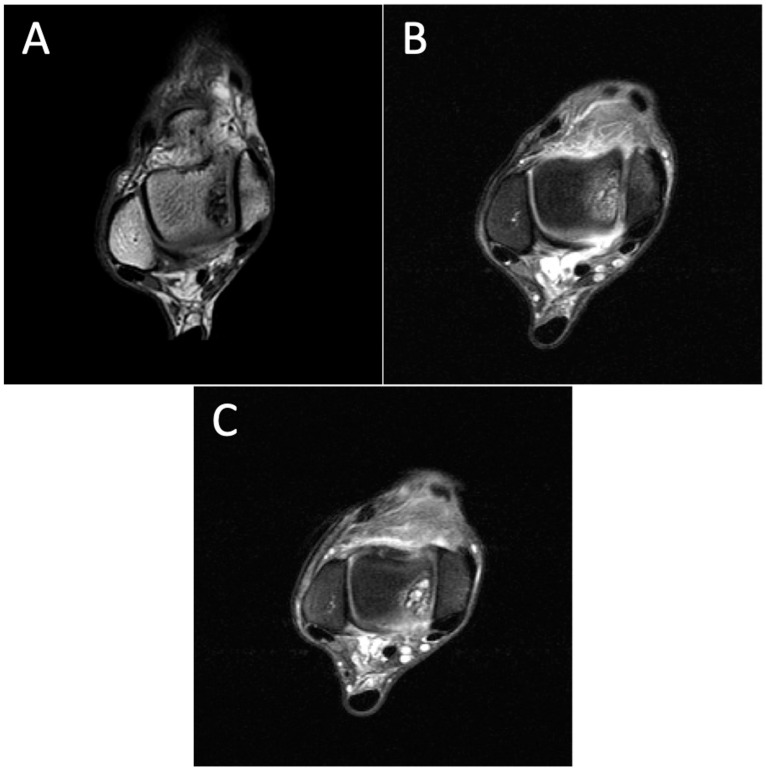
Degenerative progression of the right OLT on axial MRIs from 2011 to 2021. The timeline of OLT progression includes MRI scans from 2011 (**A**), 2015 (**B**), and 2021 (**C**).

**Figure 5:**
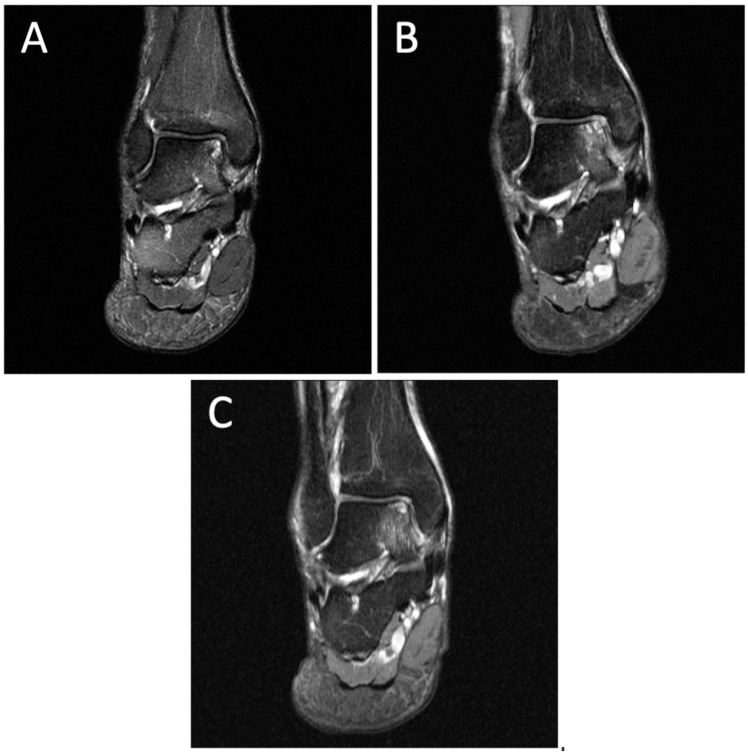
Degenerative progression of the right medial osteochondral lesion on coronal MRIs from 2011 to 2021. The timeline of OLT progression includes MRI scans from 2011 (**A**), 2015 (**B**), and 2021 (**C**).

**Figure 6:**
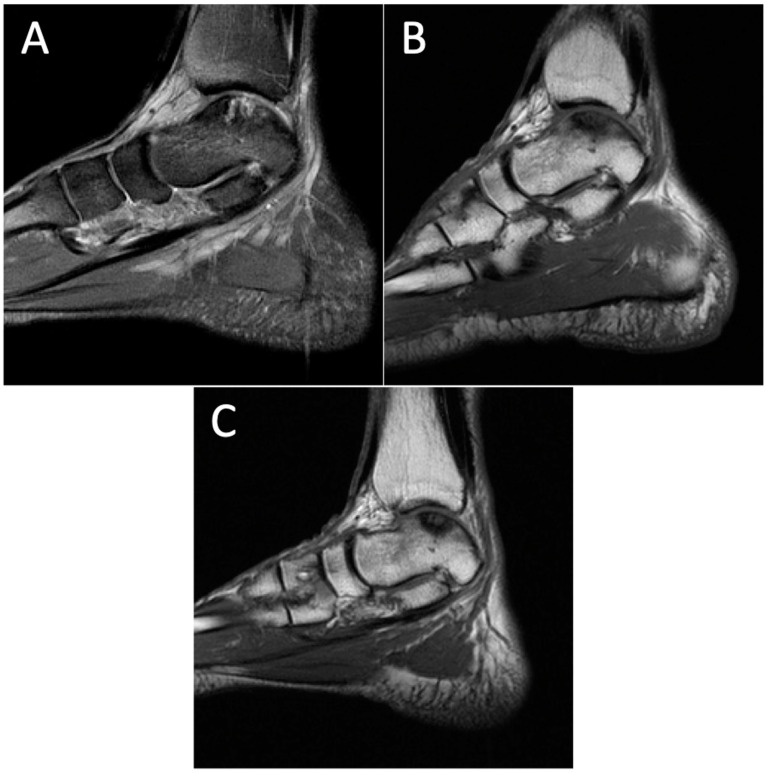
Degenerative progression of the right medial osteochondral lesion on sagittal MRIs from 2011 to 2021. The timeline of OLT progression includes MRI scans from 2011 (**A**), 2015 (**B**), and 2021 (**C**). Discussion: OLTs are common injuries that involve insult to the articular cartilage and subchondral bone of the talus. OLTs are typically trauma-related, and radiographs are often negative in the period immediately following injury. Magnetic resonance imaging (MRI) is the best imaging modality to visualize cartilage defects, soft tissue changes, and bony edema with no exposure to radiation [1,2]. As a result, the standard of care for diagnosis has shifted towards MRI for the identification and characterization of OLTs [3]. This has been substantiated as recent imaging advancements have increased image clarity with high-resolution 3.0T scanners, resulting in the improved identification of cartilage defects [4,5,6,7]. Verhagen et al. found that MRI had a sensitivity and specificity of 96% in a prospective trial, indicating that MRI is a significant tool in diagnosing these injuries [8]. The natural progression of OLTs has not been well reported in the literature. Understanding the pathological development of these lesions and which eventually develop osteoarthritic changes would be a valuable step in management. The existing literature is inconsistent, with some investigators reporting low rates of progression, while others believe that OLTs have poor long-term outcomes and a high risk of osteoarthritic changes [9]. The highest reported incidence of natural progression to osteoarthritis is 95% [10], while other studies have found an incidence of around one-third of their population cohort after five years [11,12,13]. Interestingly, some of the literature has found little to no osteoarthritic changes in similar long-term follow-up periods [14,15]. The inconsistency in the literature warrants more imaging studies to further characterize and understand the progression of these lesions. This study presents a series of images documenting a 16-year history of bilateral ankle pain in a 72-year-old man with initial symptoms caused by an exercise-induced injury. The series of MRIs are the first in the literature to visually illustrate the progression of OLT and the development of osteoarthritis over time. This case highlights the utility of MRI in the diagnosis and long-term monitoring of OLTs and emphasizes the need for more longitudinal imaging studies to better characterize the natural progression of these lesions. Future research should explore other imaging modalities in larger patient cohorts with diverse ages and durations of symptoms to increase the generalizability of these findings.
